# Editorial: Entering the Brave New World of ICD-11 Personality Disorder Diagnosis

**DOI:** 10.3389/fpsyt.2021.793133

**Published:** 2021-11-17

**Authors:** Bo Bach, Antonella Somma, Jared W. Keeley

**Affiliations:** ^1^Center for Personality Disorder Research (CPDR), Psychiatric Research Unit, Slagelse Psychiatric Hospital, Slagelse, Denmark; ^2^School of Psychology, Vita-Salute San Raffaele University, Milan, Italy; ^3^Psychology Department, Virginia Commonwealth University, Richmond, VA, United States

**Keywords:** ICD-11, classification, personality functioning, diagnosis, personality disorder, personality trait, personality disorder severity, clinical utility

## Introduction

**Figure 1:**
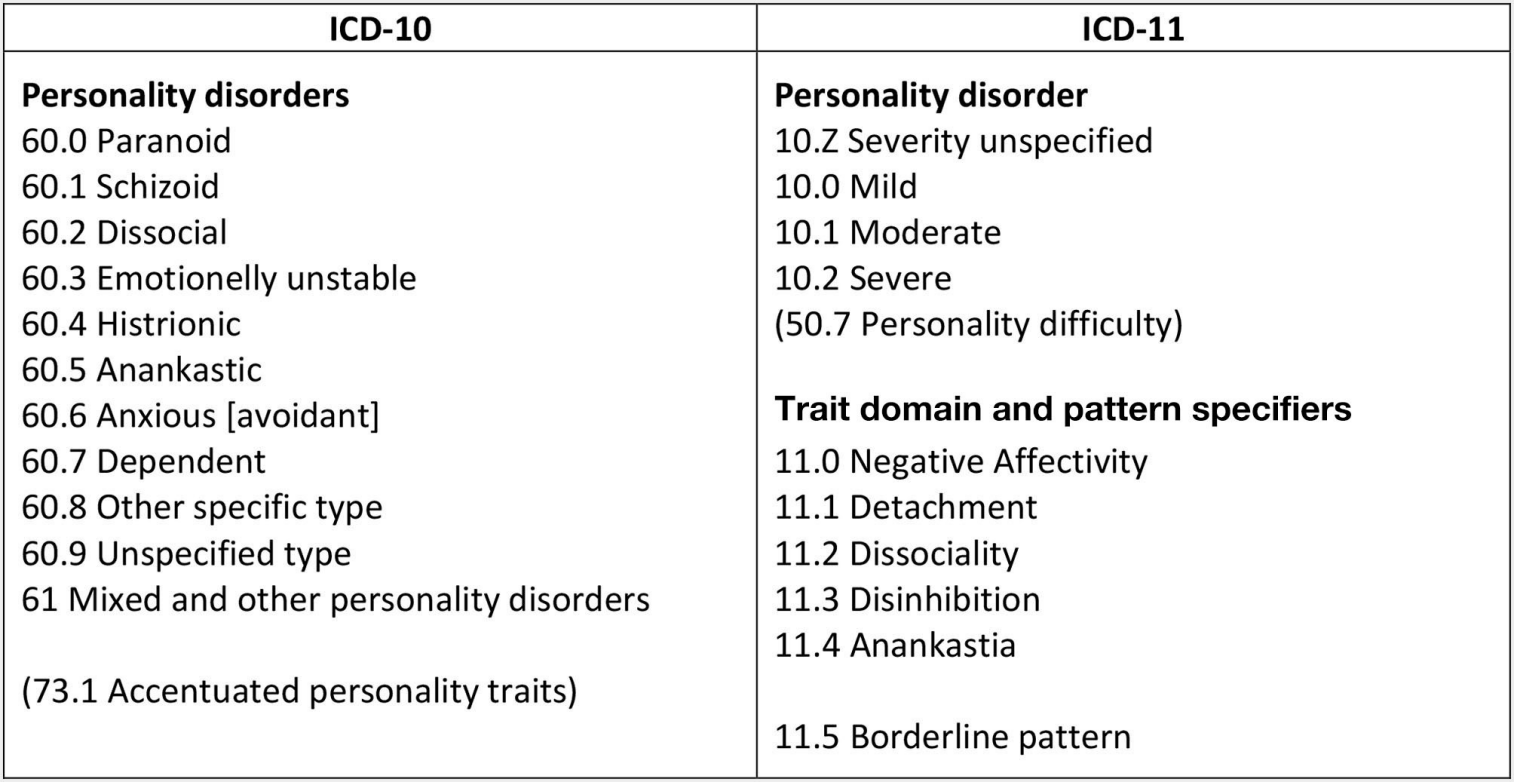
Migration from ICD-10 to ICD-11 classification of personality disorders.

WHO member states are soon expected to migrate from the ICD-10 to the ICD-11 Classification of Mental Disorders (1), which must be used for different coding purposes in mental health care including national statistics and billing for health insurance companies (see Figure 1). While most diagnoses remain unchanged^1^, a fundamentally new approach to the classification of personality disorders (PD) is introduced (1, 2). Rather than diagnosing PDs according to familiar categorical types, the clinician is now requested to focus on general impairments of self- and interpersonal functioning, along with their cognitive, emotional, and behavioral manifestations, which can be classified according to their overall severity (i.e., *Mild Personality Disorder, Moderate Personality Disorder, Severe Personality Disorder*). Otherwise, the clinician can assign a sub-diagnostic *Personality Difficulty* code (akin to ICD-10 Z73.1 accentuated personality traits). The clinician is also allowed to assign one or more trait domain specifiers that contribute to the individual expression of personality disturbances (i.e., *Negative Affectivity, Detachment, Dissociality, Disinhibition, Anankastia*). Finally, with the aim of facilitating the identification of individuals who may respond to established treatments, a *Borderline Pattern specifier* has been included, which is essentially based on the DSM-5 Borderline PD diagnostic criteria. For a more detailed historical account and rationale behind the ICD-11 PD model, we refer to the overview article by Mulder in this special topic collection.

Given this radical shift in diagnostic practice, we now take the opportunity to focus on initial and preliminary findings and considerations related to the utility of the ICD-11 classification of PDs. The 17 articles included in this special topic collection are written by authors from 16 different nations. They address various aspects of this new diagnostic approach including assessment of personality functioning, utility of trait domain specifiers, the inclusion of a separate Anankastia domain, and conceptual considerations with reference to narrative identity, mentalization, and psychodynamic theory. Apart from pointing out key findings of the articles, we will also highlight challenges and opportunities that arise with respect to operationalization, clinical implementation, and future directions.

## Assessment of Disturbances in Personality Functioning

Hutsebaut et al. show that the Semi-Structured Interview for DSM-5 Personality Functioning (STiP-5.1) may be employed to assess level of PD severity in incarcerated patients with good inter-rater reliability. Gamache et al. demonstrate that the 24-item Self- and Interpersonal Functioning Scale (SIFS) is a useful measure for determining severity of personality pathology based on the ICD-11 model, and showed promising alignment with external criteria. Finally, Clark et al. introduce a set of preliminary self-report scales for ICD-11 personality disorder, covering both personality functioning and trait domains, which generally showed excellent psychometric qualities.

## Aspects of Psychodynamic Theory, Narrative Identity, and Mentalization

Blüml and Doering discuss how the ICD-11 classification of PD severity converges with long-standing psychodynamic conceptualizations of personality pathology, and provide a meaningful common ground for assessment and treatment of PDs. Using an empirical approach, Nazari et al. show that a pan-theoretical approach to personality functioning, such as the ICD-11 classification of PD severity, is well-aligned with the object-relations model of personality functioning. Lind illustrates how narrative identity contributes an indispensable aspect to ICD-11's definition of functioning in aspects of the self that revolves around “stability and coherence of one's sense of identity.” Finally, with their empirical findings, Rishede et al. make a compelling proposal about how the capacity for mentalizing is involved in the ICD-11 model of personality functioning, which is deemed particularly relevant for its clinical utility.

## Trait Domain Specifiers

Riegel et al. overall supported the ability of the 36-item Personality Inventory for DSM-5 and ICD-11 Brief Form Plus (PID5BF+M) to capture the five ICD-11 trait domains in a Czech-Speaking community sample, with the exception of the Disinhibition domain. Pires et al. demonstrate the ability of ICD-11 trait domain specifiers to differentiate patients with PDs from other clinical groups using the PID5BF+M measure. Fang et al. affirm that the ICD-11 trait domains have acceptable psychometric features in a large Chinese sample, including structural validity and continuity with familiar PD types. Gutiérrez et al. show that four trait domains (i.e., Negative affectivity, Detachment, Dissociality/Antagonism, and Disinhibition) are roughly interchangeable across ICD-11 and DSM-5 trait systems, and propose how the trait domains' discriminant validity can be improved. Using a mixed sample from the Kurdistan region, Hemmati et al. demonstrate that the ICD-11 trait model outperforms the DSM-5 trait model in terms of factorial model fit within this particular region. Finally, Kerber et al. examine the utility of ICD-11 traits (as operationalized with the PID5BF+M) for predicting treatment outcome and serving as a measure of change.

## Rationale of Including a Separate Domain of Anankastia?

The ICD-11 PD workgroup decided to include a separate domain of Anankastia rather than focusing on low levels of Disinhibition as in the DSM-5 model. Strengths and weaknesses of this decision have already been discussed in the literature (3–7). Gecaite-Stonciene et al. review the empirical literature on personality features corresponding to the ICD-11 domain of Anankastia, and find this domain to have structural validity and substantial overlap with established traits of obsessive-compulsive PD. Using empirical data, Strus et al. show that the ICD-11 Anankastia domain reveals more distinct features of personality pathology than the DSM-5 domain of Psychoticism. Along the same lines, Clark et al. show that Anankastia is not merely the opposite end of a Disinhibition dimension. Finally, Bastiaens et al. use clinical cases to illustrate how the separate ICD-11 domain of Anankastia contribute with distinct and relevant patient information that is not merely explained by reversed Disinhibition.

## Current Approaches to the Operationalization of ICD-11 Personality Disorder Features

Clinicians across all WHO member countries should be able to diagnose a PD using the freely accessible ICD-11 Clinical Descriptions and Diagnostic Guidelines (1) *per se* without having to use additional instruments or measures. Thus, it should be doable for practitioners to determine PD severity based on clinical observations or other available material. Nevertheless, standardized instruments or measures are often indispensable for ensuring sufficient reliability.

The international community of researchers and clinicians may consider using a range of instruments of which some are specifically developed for capturing the fullness of ICD-11 Personality Disorders and Related Traits. Some of these measures and instruments were also applied in the 17 studies of this special topic collection.

For the overall assessment of PD severity, researchers and clinicians may use the 14-item Personality Disorder Severity-ICD-11 (PDS-ICD-11) scale (8), which is currently being adapted to a clinician-rating form. As highlighted in this special topic, a preliminary tool by Clark et al. may also be used to measure specific ICD-11 features of self- and interpersonal functioning by means of 65 designated items. In comparison, the STiP 5.1 structured interview used by Hutsebaut et al., the 24-item SIFS measure used by Gamache et al., and the 80-item LPFS-SF measure used by Nazari et al. may be employed to capture more general features of self- and interpersonal functioning that are relevant but not specific for the ICD-11 model. Nevertheless, of the aforementioned instruments of PD severity, only the PDS-ICD-11 scale (8) seems to account for emotional, cognitive, and behavioral manifestations, which are used in the ICD-11 to determine PD severity with respect to self-harm, reality testing (e.g., psychotic-like perceptions), and risk of harm.

For the assessment of trait domain specifiers, researchers and clinicians may consider using the new scales developed by Clark et al. to capture the specified features of ICD-11 trait domains. Practitioners may also employ the 60-item Personality Inventory for ICD-11 (PiCD) (9–13) as done in the studies by Gutiérrez et al. and Strus et al. Notably, the PiCD is also available as an informant-report form (4, 14) aimed at clinicians or relatives who know the patient well. The 121-item Five-Factor Inventory for ICD-11 (FFiCD) (15) is available for practitioners who desire a more fine-granted portrait of their patient in terms of 20 facets and 47 nuances. Moreover, the more feasible 17-item Personality Assessment Questionnaire for ICD-11 personality trait domains (PAQ-11) (16) may be particularly useful for research purposes and clinical screening.

In addition to these ICD-11-specific measures, empirically validated algorithms for the Personality Inventory for DSM-5 (PID-5) may also be used to capture the ICD-11 traits (17, 18), as done in the studies by Fang et al. and Hemmati et al. Finally, as shown in the studies by Bastiaens et al., Kerber et al., Pires et al., and Riegel et al., the 36-item PID5BF+M can be used measure essential features of both ICD-11 and DSM-5 trait domains including 18 subfacets (3, 19).

## Future Directions in Clinical Implementation and Research

Extensive knowledge is already available about the clinical utility of PD severity and dimensional assessment in general (20–22), but we only have sparse information about the specific ICD-11 definition of personality dysfunction (23). Based on comparable indices of PD severity, we expect that the introduction of the ICD-11 model may help in clinical decision making including relevant allocation of treatment resources (e.g., length, type, and intensity of treatment) (21, 24). Such an approach to allocation of resources may, if successful, help ensure treatment for those who need it the most rather than exclusively basing such decisions on individual practitioners' opinions or ideas. More studies are also needed to determine the prognostic value of classifying patients according to severity.

It also seems highly relevant to provide clinical guidelines for trait domain specifiers (in combination with the severity classification) in order to assist individualized case formulation, treatment planning, and intervention. Initial research suggests that such aspects of the ICD-11 classification's clinical utility are satisfactorily supported (21, 25–28).

Finally, we acknowledge the inclusion of a borderline pattern specifier as a preliminary pragmatic solution to divergent positions, which also may ease the transition for patients who have already been granted support or treatment based on a borderline diagnosis. Yet, preliminary research suggests that global severity of personality dysfunction substantially accounts for the variance described by Borderline PD (29–32). The ICD-11 classification of PD severity may specifically capture borderline features such as maladaptive identity functioning, poor emotion regulation, impaired reality testing under stress, and risk of harm to self (8). In addition, clinical research and meta-analytic evidence suggest that trait domains of Negative Affectivity (e.g., emotional lability), Disinhibition (e.g., impulsivity), and Dissociality (e.g., aggression) elucidate the heterogeneity of Borderline PD (33, 34).

## Author Contributions

BB wrote the first draft of the manuscript. All authors contributed to manuscript revision, read, and approved the submitted version.

## Conflict of Interest

JK and BB are involved in work related to ICD-11 field trials. The remaining author declares that the research was conducted in the absence of any commercial or financial relationships that could be construed as a potential conflict of interest.

## Publisher's Note

All claims expressed in this article are solely those of the authors and do not necessarily represent those of their affiliated organizations, or those of the publisher, the editors and the reviewers. Any product that may be evaluated in this article, or claim that may be made by its manufacturer, is not guaranteed or endorsed by the publisher.

## Footnotes

^1^ICD-11 also introduces a new classification of Autism Spectrum Disorder where the clinician can specify the degree of impairment on the spectrum rather than different types of autism; and clinicians are also allowed to use dimensional specifier scales for symptomatic manifestations of Primary Psychotic Disorders.

## References

[1] WHO. ICD-11 Clinical Descriptions and Diagnostic Guidelines for Mental and Behavioural Disorders. Geneva: World Health Organisation (2021). Available online at: gcp.network/en/private/icd-11-guidelines/disorders

[2] Reed GM. Progress in developing a classification of personality disorders for ICD-11. World Psychiatry. (2018) 17:227–8. 10.1002/wps.20533 29856549 PMC5980531

[3] Bach B Kerber A Aluja A Bastiaens T Keeley JW Claes L. International Assessment of DSM-5 and ICD-11 personality disorder traits: toward a common nosology in DSM-5.1. Psychopathology. (2020) 53:179–88. 10.1159/000507589 32369820

[4] Bach B Christensen S Kongerslev MTMT Sellbom M Simonsen E. Structure of clinician-reported ICD-11 personality disorder trait specifiers. Psychol Assess. (2020) 32:50–9. 10.1037/pas0000747 31328934

[5] Aluja A Sayans-Jiménez P García LF Gutierrez F. Location of International Classification of Diseases−11th Revision and Diagnostic and Statistical Manual of Mental Disorders, Fifth Edition, dimensional trait models in the alternative five-factor personality space. Personal Disord Theory Res Treat. (2021) 12:127–39. 10.1037/per0000460 33630629

[6] Crego C Widiger TA. The convergent, discriminant, and structural relationship of the DAPP-BQ and SNAP with the ICD-11, DSM−5, and FFM trait models. Psychol Assess. (2020) 32:18–28. 10.1037/pas0000757 31328932

[7] McCabe GA Widiger TA. A comprehensive comparison of the ICD-11 and DSM−5 section III personality disorder models. Psychol Assess. (2020) 32:72–84. 10.1037/pas0000772 31580095

[8] Bach B Brown TA Mulder RT Newton-Howes G Simonsen E Sellbom M. Development and initial evaluation of the ICD-11 personality disorder severity scale: PDS-ICD-11. Personal Ment Health. (2021) 15:223–36. 10.1002/pmh.1510 34002530

[9] Carnovale M Sellbom M Bagby RM. The Personality Inventory for ICD-11: Investigating reliability, structural and concurrent validity, and method variance. Psychol Assess. (2020) 32:8–17. 10.1037/pas0000776 31556679

[10] Oltmanns JR Widiger TA. A self-report measure for the ICD-11 dimensional trait model proposal: The Personality Inventory for ICD-11. Psychol Assess. (2018) 30:154–69. 10.1037/pas0000459 28230410 PMC5930359

[11] Somma A Gialdi G Fossati A. Reliability and construct validity of the Personality Inventory for ICD-11 (PiCD) in Italian adult participants. Psychol Assess. (2020) 32:29–39. 10.1037/pas0000766 31414851

[12] Tarescavage AM Menton WH. Construct validity of the personality inventory for ICD-11 (PiCD): Evidence from the MMPI-2-RF and CAT-PD-SF. Psychol Assess. (2020) 32:889–95. 10.1037/pas0000914 32525344

[13] Gutiérrez F Aluja A Ruiz J García LF Gárriz M Gutiérrez-Zotes A. Personality disorders in the ICD-11: Spanish validation of the PiCD and the SASPD in a mixed community and clinical sample. Assessment. (2020). 10.1177/1073191120936357 32583685 PMC7961637

[14] Oltmanns JR Widiger TA. The self- and informant-personality inventories for ICD-11: agreement, structure, and relations with health, social, and satisfaction variables in older adults. Psychol Assess. (2021) 33:300–10. 10.1037/pas0000982 33779193 PMC8483599

[15] Oltmanns JR Widiger TA. The Five-Factor Personality Inventory for ICD-11: a facet-level assessment of the ICD-11 trait model. Psychol Assess. (2020) 32:60–71. 10.1037/pas0000763 31414852 PMC6928398

[16] Kim Y-R Tyrer P Hwang S. Personality Assessment Questionnaire for ICD-11 personality trait domains: development and testing. Personal Ment Health. (2021). 15:58–71 10.1002/pmh.1493 32638542

[17] Bach B Sellbom M Kongerslev MT Simonsen E Krueger RF Mulder RT. Deriving ICD-11 personality disorder domains from dsm-5 traits: initial attempt to harmonize two diagnostic systems. Acta Psychiatr Scand. (2017) 136:108–17. 10.1111/acps.12748 28504853

[18] Sellbom M Solomon-Krakus S Bach B Bagby RM. Validation of Personality Inventory for DSM−5 (PID-5) algorithms to assess ICD-11 personality trait domains in a psychiatric sample. Psychol Assess. (2020) 32:40–9. 10.1037/pas0000746 31204821

[19] Kerber A Schultze M Müller S Rühling RM Wright AGC Spitzer C. Development of a Short and ICD-11 Compatible Measure for DSM-5 Maladaptive Personality Traits Using Ant Colony Optimization Algorithms. Assessment. (2020). 10.1177/1073191120971848. [Epub ahead of print]. 33371717 PMC8866743

[20] Crawford MJ Koldobsky N Mulder RT Tyrer P. Classifying personality disorder according to severity. J Pers Disord. (2011) 25:321–30. 10.1521/pedi.2011.25.3.321 21699394

[21] Bach B Simonsen S. How does level of personality functioning inform clinical management and treatment? Implications for ICD-11 classification of personality disorder severity. Curr Opin Psychiatry. (2021) 34:54–63. 10.1097/YCO.0000000000000658 33252430

[22] Keeley JW Briken P Evans SC First MB Klein V Krueger RB. Can clinicians use dimensional information to make a categorical diagnosis of paraphilic disorders? An ICD-11 Field Study. J Sex Med. (2021) 18:1592–606. 10.1016/j.jsxm.2021.06.016 34373211

[23] Bach B Mulder RT. Empirical Foundation of the ICD-11 classification of personality disorders. In: Huprich SK, editor. Personality Disorders and Pathology: Integrating Clinical Assessment and Practice in the DSM-5 and ICD-11 Era. 2nd ed. Washington, DC: American Psychological Association (in press).

[24] Mulder RT Bach B. Assessment and treatment within the ICD-11 framework. In: Huprich SK, editor. Personality Disorders and Pathology: Integrating Clinical Assessment and Practice in the DSM-5 and ICD-11 Era. 2nd ed. Washington, DC: American Psychological Association (in press).

[25] Morey LC Benson KT. Relating DSM-5 section II and section III personality disorder diagnostic classification systems to treatment planning. Compr Psychiatry. (2016) 68:48–55. 10.1016/j.comppsych.2016.03.010 27234182

[26] Tracy M Tiliopoulos N Sharpe L Bach B. The clinical utility of the ICD-11 classification of personality disorders and related traits: a preliminary scoping review. Aust New Zeal J Psychiatry. (2021) 55:849–62. 10.1177/00048674211025607 34144646

[27] Hansen SJ Christensen S Kongerslev MT First MB Widiger TA Simonsen E. Mental health professionals' perceived clinical utility of the ICD-10 vs. ICD-11 classification of personality disorders. Personal Ment Health. (2019) 13:84–95. 10.1002/pmh.1442 30989832

[28] Bach B Tracy M. Clinical utility of the AMPD: a 10th year anniversary review. Personal Disord Theory Res Treat. (2021). 10.1037/per0000527 35787123

[29] Sharp C Wright AGC Fowler JC Frueh BC Allen JG Oldham J. The structure of personality pathology: both general (‘g') and specific (‘s') factors? J Abnorm Psychol. (2015) 124:387–98. 10.1037/abn0000033 25730515

[30] Wright AGC Hopwood CJ Skodol AE Morey LC. Longitudinal validation of general and specific structural features of personality pathology. J Abnorm Psychol. (2016) 125:1120–34. 10.1037/abn0000165 27819472 PMC5119768

[31] Clark LA Nuzum H Ro E. Manifestations of personality impairment severity: comorbidity, course/prognosis, psychosocial dysfunction, and ‘borderline' personality features. Curr Opin Psychol. (2018) 21:117–21. 10.1016/j.copsyc.2017.12.004 29291458

[32] Mulder RT Horwood LJ Tyrer P. The borderline pattern descriptor in the International Classification of Diseases, 11th Revision: a redundant addition to classification. Aust New Zeal J Psychiatry. (2020) 54:1095–100. 10.1177/0004867420951608 32900208

[33] Bach B Sellbom M Skjernov M Simonsen E. ICD-11 and DSM-5 personality trait domains capture categorical personality disorders: finding a common ground. Aust New Zeal J Psychiatry. (2018) 52:425–34. 10.1177/0004867417727867 28835108

[34] Watters CA Bagby RM Sellbom M. Meta-analysis to derive an empirically based set of personality facet criteria for the alternative DSM-5 model for personality disorders. Personal Disord Theory Res Treat. (2019) 10:97–104. 10.1037/per0000307 30520649

